# Supplementary material for: (–)-Epicatechin Improves Vasoreactivity and Mitochondrial Respiration in Thermoneutral-Housed Wistar Rat Vasculature
Source: Nutrients. 2022 Mar 5;14(5):1097. doi: 10.3390/nu14051097 (PMC8912787; doi:10.3390/nu14051097)

Supplementary Figure S1A: Blots 1 and 2, total protein image for peNOS, eNOS, and SIRT1.

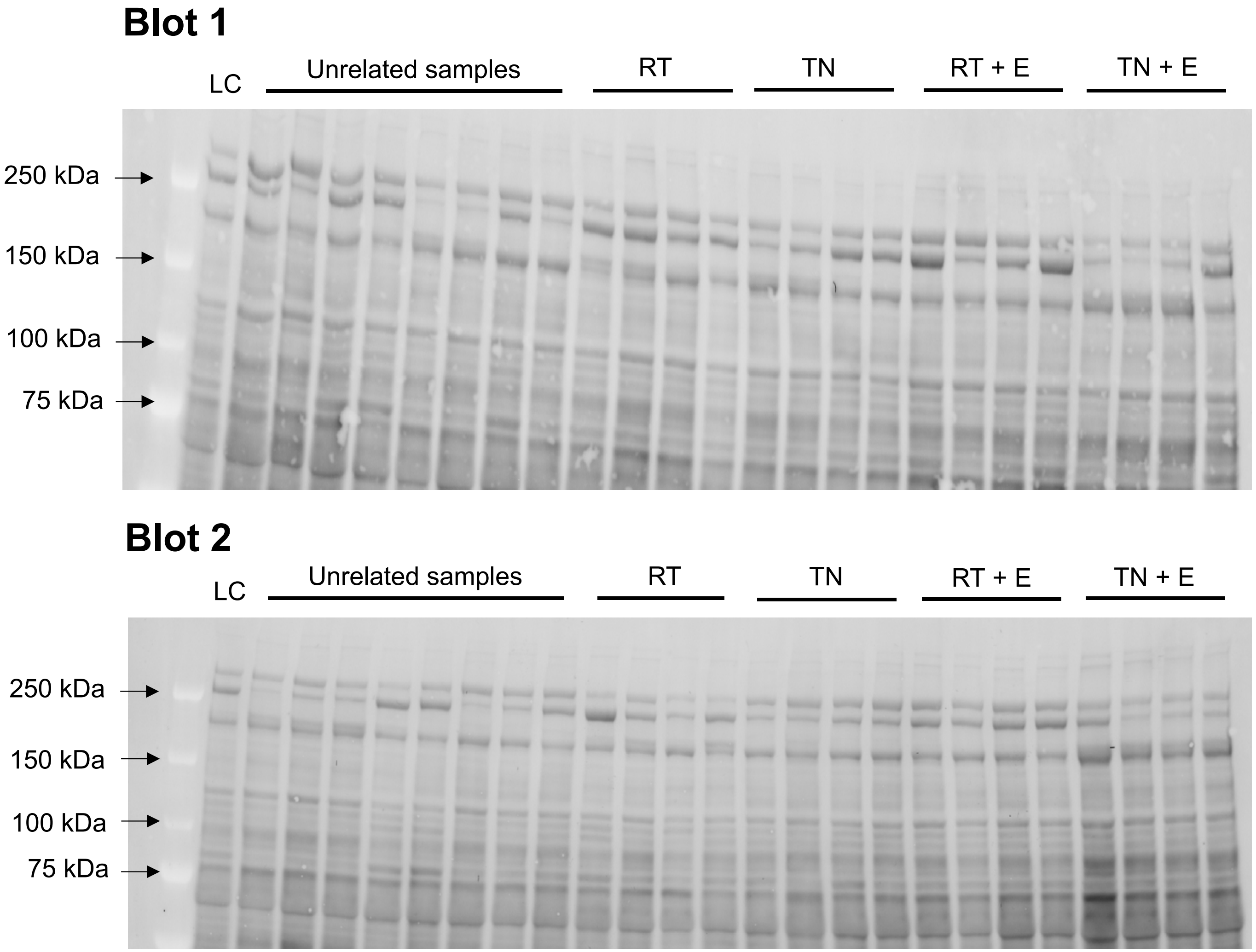

Supplementary Figure S1B: Blots 3 and 4, total protein image for pAMPK, AMPK, MnSOD, PGC1-α and mitochondrial complexes.

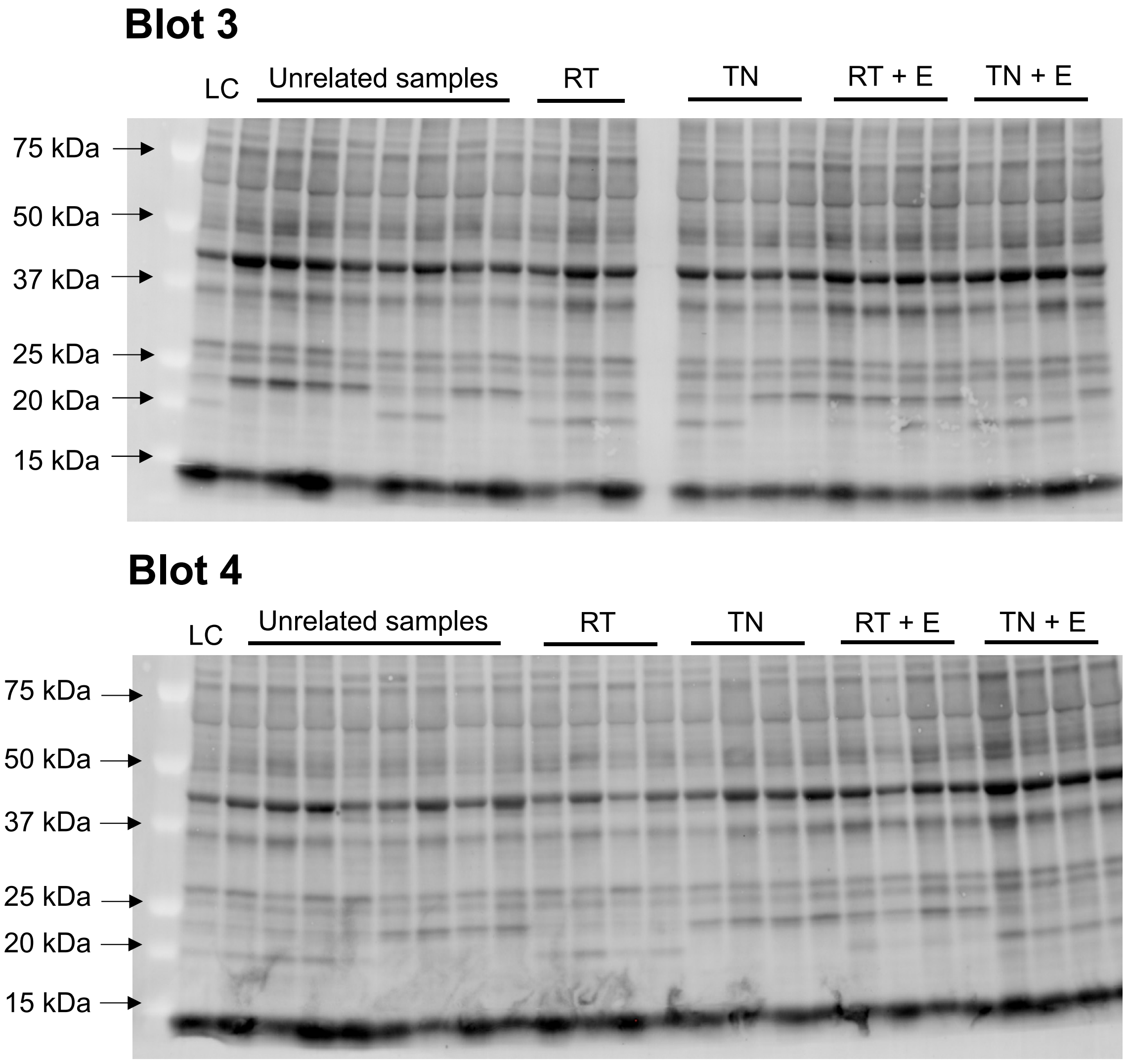

Supplementary Figure S1C: Blots 5 and 6, total protein image for SIRT3, pCaMKII, CaMKII, catalase, and SOD.

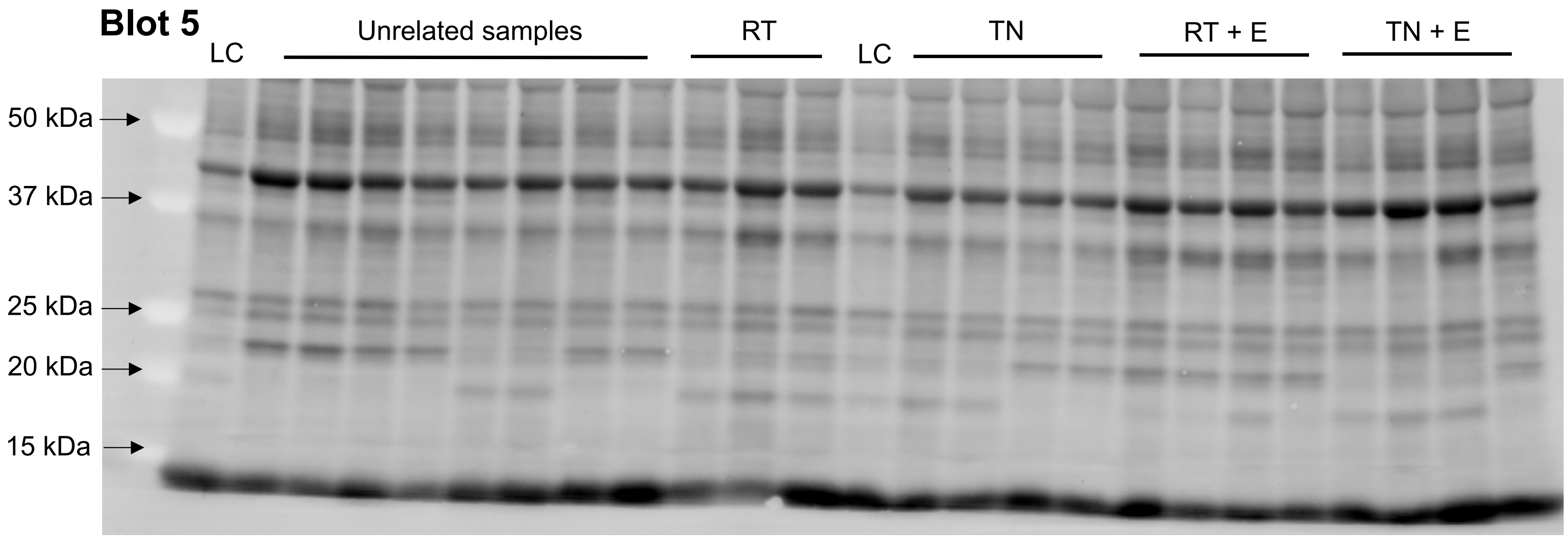

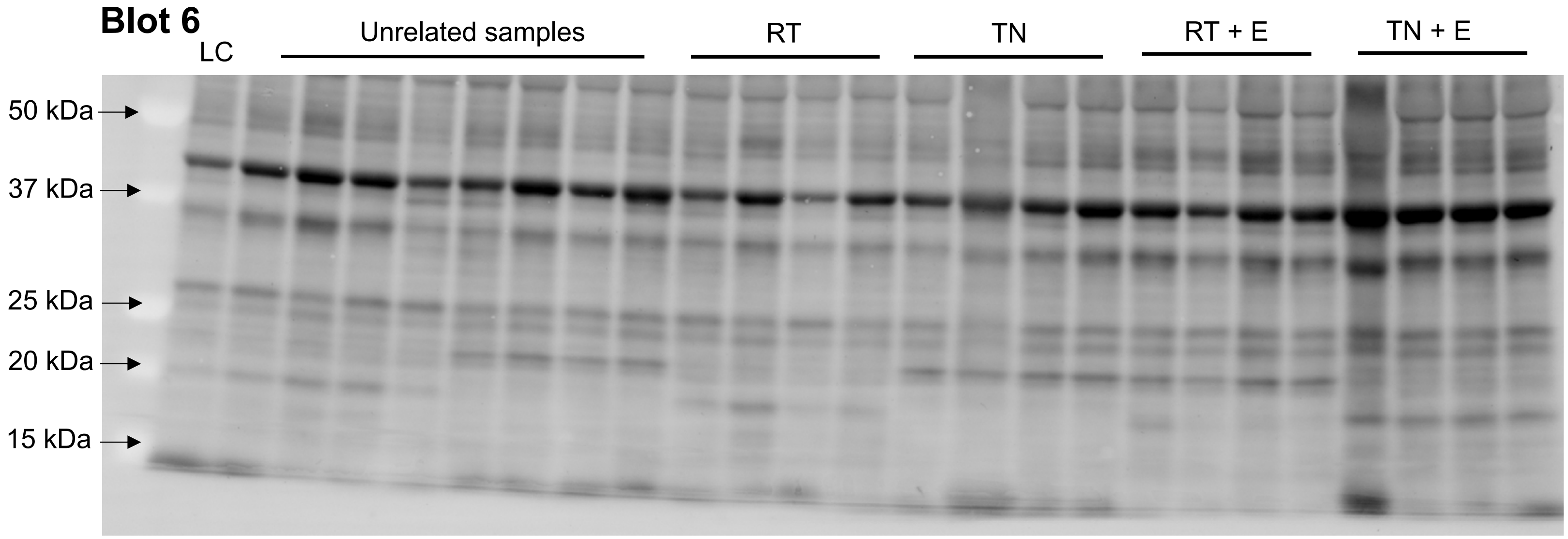

Supplementary Figure S1D: Non-denatured blots 1 and 2, total protein image for monomer/dimer eNOS.

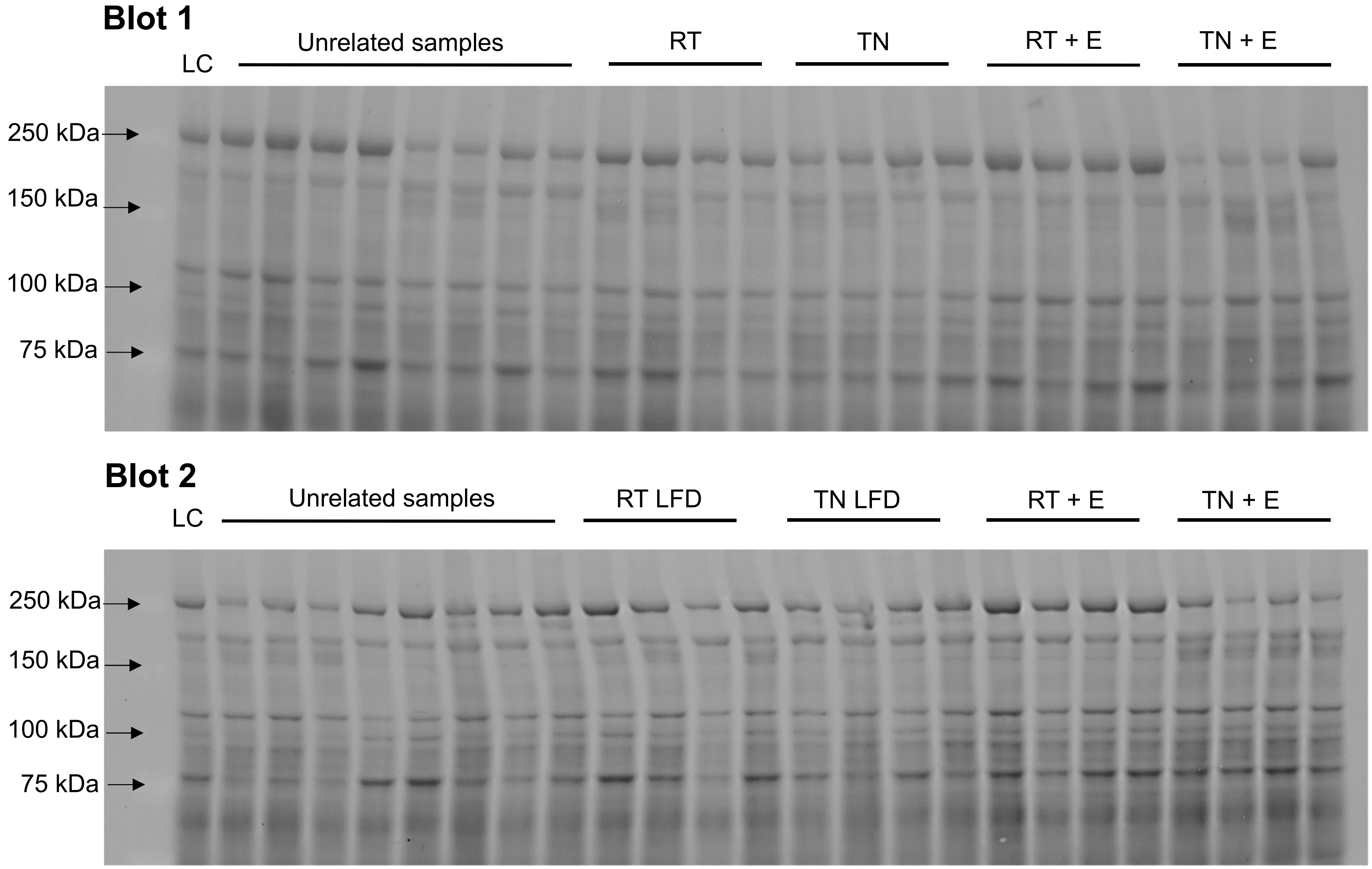

Supplement: Supplementary file 1 [file nutrients-14-01097-s001.zip › nutrients-1532178-supplementary.pdf]
